# Well-Being as a Precursor and Consequence of Micro-Processes in a Group Psychotherapy With Forensic Patients

**DOI:** 10.3389/fpsyt.2020.00409

**Published:** 2020-06-03

**Authors:** Madeleine Bieg, Thomas Ross, Jan Bulla, Tilman Kluttig, María Isabel Fontao

**Affiliations:** ^1^Department of Forensic Psychiatry and Psychotherapy, Centre for Psychiatry Reichenau, Reichenau, Germany; ^2^Department of Empirical Educational Research, University of Konstanz, Konstanz, Germany; ^3^Department of Psychosomatic Medicine and Psychotherapy, Ulm University, Ulm, Germany; ^4^Department of Forensic Psychiatry and Psychotherapy, Ulm University, Günzburg, Germany; ^5^Department of Psychology, University of Konstanz, Konstanz, Germany

**Keywords:** forensic psychotherapy, well-being, therapeutic process research, offender treatment, addiction

## Abstract

Psychotherapy is an important approach for the treatment of psychiatric disorders. Apart from treating disorders as such, psychotherapy aims at increasing patients' well-being. The Therapeutic Cycles Model (TCM) is a process-oriented theoretical model that makes predictions about the psychotherapeutic progress based on verbatim content. The model helps to identify therapeutic factors on a language level. The present study aims at analyzing transcripts of group therapy sessions with forensic psychiatric patients using the rationale of the TCM. Furthermore, the relationship between linguistic features of psychotherapy sessions and patients' well-being before and after therapy are investigated. In order to identify therapeutic factors, a group psychotherapy with nine drug addicted forensic psychiatric patients was videotaped and transcripts of *N* = 16 sessions were analyzed. Process-oriented measures were rated by the patients, their therapists, and an external observer. Patients' self-reported well-being before therapy was negatively related to *Connecting* (indicating emotional insight), and the frequency of therapeutic cycles, which are both thought of as key moments in therapy. Well-being of forensic patients is not necessarily a helpful precursor for insightful and productive events in therapy to occur. The findings help to better understand psychotherapeutic micro-processes throughout forensic therapies, and their relationship with patients' well-being. Implications for research and the forensic practice are discussed.

## Introduction

The psychotherapeutic treatment of offenders and forensic patients is effective (1–3). Yet, it is not entirely clear which psychotherapeutic processes promote behavioral change. Thus, micro-processes in psychotherapy and change agents in offender treatment are in the center of ongoing psychotherapy process-outcome research (4). A very broad and prominent model that summarizes empirical factors at play in psychotherapy is the Generic Model of Psychotherapy (5). Six aspects of psychotherapy are outlined: therapeutic contract, therapeutic operations, therapeutic bond, participant self-relatedness, in-session impacts, and temporal patterns. In-session impacts describe what happens in a therapy session, i.e. emotional reactions, or insight.

The Therapeutic Cycles Model (TCM; 6, 7) is a process-oriented theoretical model embedded in the framework of the Generic Model of Psychotherapy. Focusing on emotion and cognitive reflection of intra-psychic processes, it makes empirical assumptions and predicts progress in psychotherapy based on the verbatim content of therapy sessions. Using the rationale of the TCM, the present study aims at analyzing transcripts of group therapy sessions with forensic psychiatric patients diagnosed with substance use disorders. The focus of this study is on describing the relationships between micro-processes in psychotherapy sessions using text analysis and session ratings (by patients, therapists, and external observers).

## Theoretical Background

### Well-Being in Therapy

In forensic process-outcome research, positive outcome may be broadly defined as the improvement of psychiatric symptoms and no further delinquency. These are the hard criteria for positive treatment outcome in forensic samples. In order to reach a positive outcome, a number of intra- and interpersonal processes related to psychological and behavioral change must have occurred. For this reason, it is necessary to study more immediate “outcomes” including well-being after the therapy session. The relationship of well-being and treatment success is not entirely clear; psychotherapy with forensic patients is hard work for both the therapists and the patients. Thus, therapy may both increase and decrease patients' subjective well-being.

Well-being in therapy became a topic of interest when the positive psychology movement started. A therapeutic approach called well-being therapy was created and tested in forensic populations (8, 9). Yet, few studies investigated well-being and affect during therapy, i.e. as a precursor, and/or a consequence of therapeutic sessions in therapists and patients (10). We do not know of any in forensic populations.

### Therapeutic Factors and Micro-Processes in Therapy

A very early review by Corsini and Rosenberg (11) identified three main therapeutic factors in group therapy called intellectual (e.g., realizing that others have similar problems, intellectualization, learning), emotional (e.g., mutual help, safety within the group), and actional factors (e.g., interaction, catharsis). Expressing emotions, reflecting about one's actions and emotions, and the coherent narrative of these experiences are considered important variables of insight-oriented therapeutic interventions. This also applies to more recent conceptualizations of therapeutic agents (12–14). A very influential conceptualization of group therapeutic change agents stems from Yalom (see 15 for a concise overview of decades of research). These are universality, altruism, instillation of hope, imparting information, corrective recapitulation of the primary family experience, development of socializing techniques, imitative behavior, cohesiveness or cohesion (possibly the primary factor from which all others emanate), existential factors, catharsis, interpersonal learning, and self-understanding. There is strong evidence of a moderate relationship between group cohesion and outcome in group therapy (16). Empirical findings on Yalom's generic factors in group therapy for sex offenders were compiled in a review article (17).

It is assumed that the therapeutic factors at play do not profoundly differ between therapies for forensic and non-forensic patients. In therapies with mentally disordered offenders, the improvement of a person's ability to talk about and to regulate feelings, to reflect about experiences, and to build a coherent narrative, are seen as major therapeutic goals (18). These regulatory skills are expected to translate into pro-social behaviors and may therefore contribute to prevent further delinquency. In one of the first studies on this topic, insight and catharsis were described as important therapeutic factors for drug addicted patients (19). The same holds for forensic patients. The ability to verbalize one´s feelings and emotions is considered important with regard to a positive therapeutic process (20).

Although there is ample evidence on therapeutic factors in group therapies in general (15, 21), little is known about the micro-processes that may be associated with these factors. One process-oriented model aiming to elucidate therapeutic micro-processes during therapeutic sessions is the Therapeutic Cycles Model (TCM; 6, 22).

### The Therapeutic Cycles Model

The assessment of change agents including affective experiencing or cognitive mastery (23) comes with a number of methodological challenges. At the language level, the study of therapeutic factors is both an objective and a first step to investigate micro-processes in therapy. The Therapeutic Cycles Model (6, 22) is a process-oriented theoretical model that makes assumptions about progress in therapy based on verbal content. The model also provides a methodological framework for the assessment of basic therapeutic factors. Based on transcripts of therapeutic sessions, different patterns of verbatim content in a therapy session are identified. Basically, the *emotional tone* of a text as an indicator of an emotional event (positive and negative), and *abstraction* as a manifestation of cognitive-reflective processes are distinguished. Furthermore, *narrative style* is assessed. Based on the most prominent contents of a therapy sequence, four different Emotion–Abstraction patterns are identified. These patterns are named *Relaxing* (indicating emotional tone and abstraction measures below the mean), *Reflecting* (emotional tone below the mean and abstraction above the mean), *Experiencing* (emotional tone above the mean and abstraction below the mean), and *Connecting* (emotional tone and abstraction above the mean). *Connecting* is assumed to describe key moments of therapy, and to reflect therapeutic situations in which insight occurs. *Connecting* enables reflection about one's self, especially about one's emotions.

The prototypical cycle starts with *Relaxing* followed by a phase of intense negative emotions. Through therapeutic intervention, an increase of positive emotions is assumed (*Experiencing*). The third phase consists of a reflection process shifting the emotional pattern into *Connecting*. In phase four, emotional arousal is thought to decline as a result of insight. This is when *Reflecting* emerges. The therapeutic cycle ends with *Relaxing*. A later version of the model held that *Connecting* should be preceded, and followed by *Relaxing* (22).

The Therapeutic Cycles Model is rooted in the Resonating Minds Theory, which makes use of two concepts called “deepen-and-provide” and “broaden-and-build” (24, 25). “Deepen-and-provide” relates to a conceptual connection between negative emotional states (e.g. negative mood) and problem-centered therapeutic work, i.e. if negative emotion prevails, problem-centered psychotherapeutic work is indicated. “Broaden-and-build” relates to a positive relationship between positive emotional states (e.g. positive mood) and creative thinking and problem-solving. Thus, therapeutic interventions should take into account cognitive processes as they relate to emotional valence (24, 26, 27).

**Figure 1:**
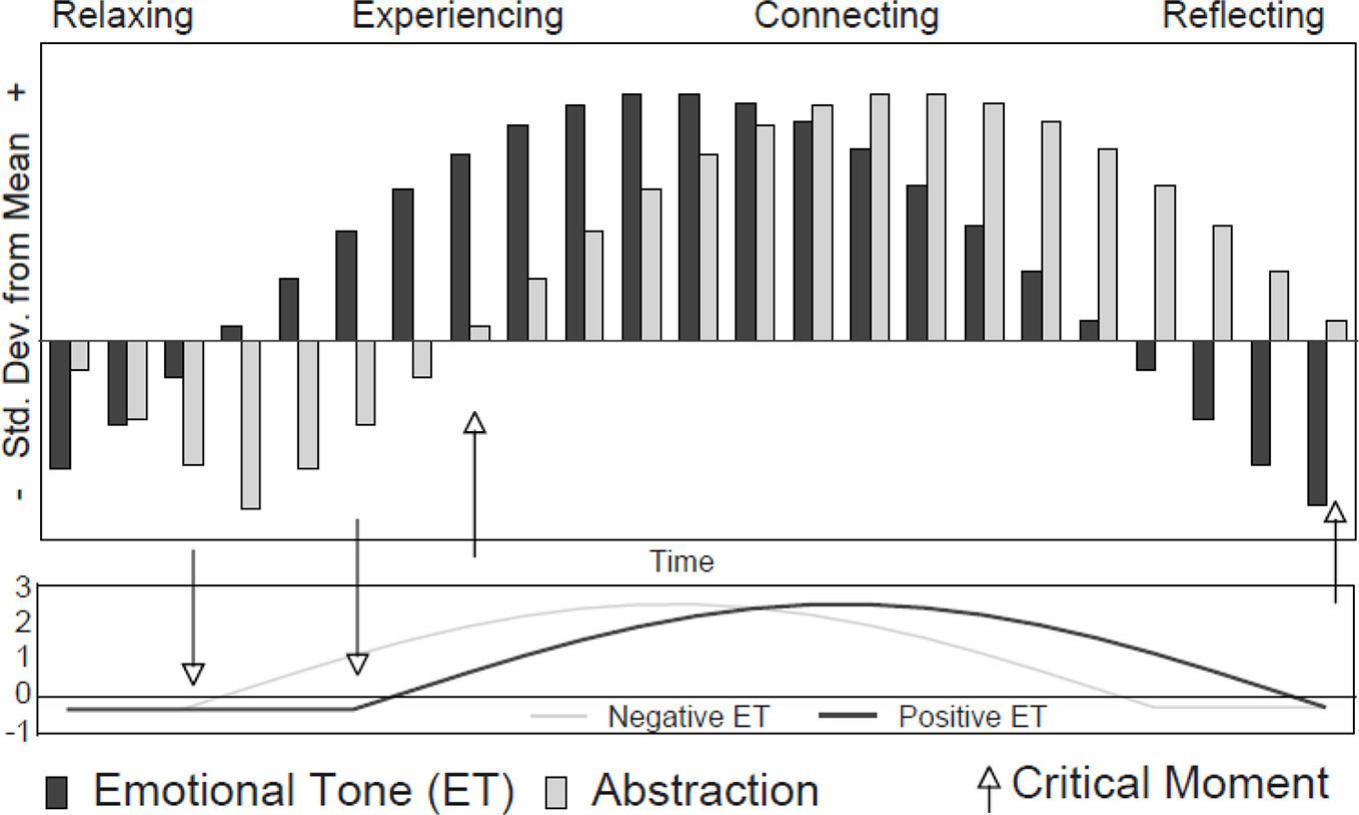
Prototypical depiction of the Therapeutic Cycles Model. (22, p. 113).

According to Mergenthaler (24), identifying critical moments in therapy is essential to helping patients progress (see arrows in **Figure 1**). Four critical moments are highlighted. First, patients may have difficulties accessing conflictual material. Second, the transition between “deepen-and-provide” and “broaden-and-build” may not work properly, and patients may be stuck in negative experiencing. Third, patients may not able to reflect their feelings. Finally, a new therapeutic cycle should start. The main therapeutic challenge for therapists consists in helping patients to successfully pass critical moments.

There are various studies on the Therapeutic Cycles Model and its relationship with other theories of therapeutic processes, therapeutic orientations, and clinical disorders (22, 24, 25, 28, 29). Importantly, TCM has successfully been adapted to the group setting (30–33). In patients whose therapies were considered successful, *Connecting* was more frequent as compared to nonimproved patients (6). In the group format, *Connecting* and insight were positively correlated (30). Hence, *Connecting* is thought to indicate key situations in therapy.

Three studies applied the TCM to the analysis of forensic therapy. Böhmer, Mergenthaler and Pfäfflin (34) conducted a single case study using the transcripts of a sexual offender in a forensic correctional setting. The patient used more words indicating positive and negative emotion than the therapist. As the therapy proceeded, there was an increase of emotionally toned and abstract words. *Connecting* was positively related to quality ratings of the therapeutic session. Pfäfflin and colleagues (20) compared cognitive–behavioral therapy sessions of sexual offenders with neurotic patients in a psychodynamic therapy. *Connecting* was found more frequent in therapies with forensic patients. However, no group differences emerged in the frequency of *Experiencing* and *Reflecting*. In the third study, three therapies of forensic patients conducted in a mental hospital were compared. In the least successful therapy, no therapeutic cycle was identified. The successful therapy showed more complete therapeutic cycles than the less successful therapies (35).

## The Present Research

The main goal of the present case study was to analyze micro-processes in a group psychotherapy with drug addicted offenders using the rationale of the TCM. Process studies on forensic inpatients are scarce; we aimed at providing insight into what happens in forensic therapy sessions. Second, the present study assessed well-being of patients immediately before and after therapy sessions; in addition, we measured how well-being changes throughout therapy. Finally, the present study examined relationships between therapeutic factors as measured on a language level using the TCM and session ratings. In addition to patients' well-being, ratings on therapy motivation, therapeutic process as evaluated by the therapists, and the productiveness of sessions were used.^1^

## Material and Methods

### Ethical Statement

The study was conducted in accordance with the Declaration of Helsinki, the local legislation, and institutional requirements. All patients provided written consent before study entry. Data were collected in the context of a pilot program aiming at developing quality assurance procedures for group treatment in a forensic setting. These included the measurement of core aspects of the delivered treatment and patient-based data on treatment satisfaction. The overarching purpose of the program was to monitor and to improve the quality of the delivered forensic treatment.

In Germany, national regulations (sec. 135a SGB V; obligation of service providers for quality assurance) stipulate that health services providers (including hospitals) must implement procedures in order to monitor and to ensure the quality of the treatments. Patients' views on treatment satisfaction and enhancing therapists' competences play a prominent role. As quality insurance is a part of the legal duties of mental health services providers, a separate ethics vote was not required to collect data for the purpose of monitoring and ensuring the quality of the delivered treatment. Patients who participated in the group therapy were informed about the aims and measures of the program. Participation was voluntary, and neither the participation nor refusing participation had any effects on the treatment, or legal consequences.

### Sample and Procedure

The data were gathered within a project aimed at controlling the quality of treatment in a forensic psychiatric clinic in South West Germany. In the years 2008 and 2009, a group psychotherapy with nine drug addicted forensic psychiatric patients was videotaped and *N* = 16 consecutive therapy sessions were transcribed. It was a high-frequency, slow-open, integrative group psychotherapy with each session lasting around 90 min. Two therapists and a nurse ran the therapy, except for four sessions, where one therapist was missing. As some patients were missing during single therapy sessions, the group composition differed between the sessions. The patients were all treated according to section 64 of the German penal code (placement in an addiction treatment facility). The patients' index offences comprised violations of the German Narcotics act, property offences, violent assaults, and other violent offences (e.g., robbery, extortion, coercion), attempted homicide, and homicide. The patients had a mean age of *M* = 33 years (range between 22 and 47 years). One patient had migrated to Germany. The mean time spent in the clinic was *M* = 6.2 months (range between 0 and 13 months). The patients were diagnosed with ICD-10 diagnoses of mental and behavioral disorders due to psychoactive substance use (ICD-10: F10-F19). Some patients had other comorbid psychiatric disorders. The therapy sessions were videotaped and transcribed. The process-oriented measures were rated by the patients and the therapists before and after therapy.

### Assessments and Measures

The verbatim transcripts were analyzed using computer-assisted text analysis. Dictionaries of the vocabulary denoting emotional tone, abstraction, and narrative style are available. The program CM (37) was used to compare the transcripts with entries in the dictionaries.

The emotional tone dictionary comprised 13,541 words. Verbs, adjectives, and adverbs relevant to emotional experience, cognitive appraisal, emotional relation, and surprise, are listed in the emotional tone dictionary. Sample words are “enthused”, “bored”, “accept”, “disdain”, “love”, “lonely”, “puzzled”. The abstraction dictionary comprised 14,187 words (only nouns). These are easily identified by their typical endings, e.g. -ity or -ness. In the emotional tone-abstraction dictionary both aspects come together in one word. Words of this category are thought to contain aspects of emotion *and* abstraction. Sample words are “enjoyment”, “tenderness”, “contemptuousness”, “distance”. The narrative style dictionary contains typical words found in narratives.

First, checks on emotional tone, abstraction, and narrative style were applied on all words that did not show up in the dictionaries. If suitable, they were added to the dictionaries. For micro-analysis of individual therapy sessions, the frequency of emotional and abstract words was analyzed in blocks of 200 words. Frequencies were *z*-standardized in order to identify the TCM pattern. Finally, relative frequencies (= absolute frequency of the TCM pattern divided by 200 word blocks per session) were calculated.

Within the segments, the relative frequencies of emotional tone, abstraction, and narrative style, were calculated. Based on their *z*-standardized values, the patterns were determined: *Relaxing* (emotional tone and abstraction ≤0), *Reflecting* (emotional tone ≤0 and abstraction >0), *Experiencing* (emotional tone >0 and abstraction ≤0), and *Connecting* (emotional tone and abstraction >0). Furthermore, it was determined whether a word block was part of a therapeutic cycle. This is the case when *Connecting* takes place between phases of *Relaxing* and emotional tone and abstraction >0.25.

### Session Ratings by Patients, Therapists, and External Rater

For session ratings, patients and therapists marked their answers on visual analogue scales. The patients rated their subjective well-being before and after each therapy session *(“At the moment, I feel very bad … very good”)*. Furthermore, patients' motivation to attain the therapy session was assessed *(“My motivation to attend therapy today was very low … very high.”*). The therapists rated the quality of the therapeutic process for each therapy session (*“The therapeutic progress in the present session was insufficient … excellent.”*).

Furthermore, the videotaped group sessions were rated by an external observer (a psychology student in her final year of study) using the Kiel Group Psychotherapy Process Scale (KGPPS; 38). The KGPPS comprises four scales with a total of 57 items and can be retrieved online (39). Each item is rated on a five-point Likert scale. Scale I allows for a rating of the whole session (global impression of the session, productiveness of the session, the degree to which group members get along with each other, non-constructive use of silence, real interaction between patients and therapists). For the present study, we used the productiveness rating of Scale I because productiveness can effectively be compared to the analyses of micro-processes on a language level. According to the manual, productiveness is high when the therapeutic work appears to be effective during the session, i.e. if single group members make progress. The interrater reliability was satisfactory. For 16 sessions, only two sessions differed by more than one rating point.

### Statistical Analyses

The patients' and therapists' scores were averaged. As language variables were not normally distributed, nonparametric statistics were applied where appropriate. Friedman tests for multiple paired samples and Wilcoxon signed-rank tests for paired samples were applied. T-tests for normally distributed variables were applied. Where appropriate, Spearman rank correlations were calculated.

## Results

### Therapeutic Factors on the Language Level

The total text comprised 202,281 words with 99,073 words spoken by the patients and 103,208 words spoken by the therapists. The 16 therapy sessions were divided into 1,010 segments. On average, 63 segments per transcript were extracted (range between 51 and 72). **Table 1** presents language variables for the total text, patients' text, and therapists' text. Emotional tone and abstraction as well as positive emotion-abstraction words were more frequent in the therapists' text. Narrative style was higher in the patients' text. Generally, the patients' text did not contain much emotional tone.

**Table 1:** Language variables for the total text, and separately for patients and therapists.

Language variables	Total text	Patients	Therapists	Wilcoxon-Test mean level difference between p and t
	***M (SD)***	***M (SD)***	***M (SD)***	***p***
**Emotional tone**	.044 (0.018)	.041 (0.049)	.047 (0.038)	<.001***
**Abstraction**	.062 (0.020)	.051 (0.037)	.066 (0.037)	<.001***
**Narrative Style**	.219 (0.039)	.217 (0.085)	.196 (0.076)	<.001***
**Positive emot. tone**	.021 (0.013)	.019 (0.034)	.023 (0.029)	<.001***
**Negative emot. tone**	.023 (0.014)	.022 (0.029)	.024 (0.025)	<.002**
**Positive emotion-abstraction words**	.004 (0.006)	.004 (0.011)	.005 (0.010)	<.001***
**Negative emotion-abstraction words**	.007 (0.007)	.006 (0.011)	.008 (0.014)	.031*

Segmentation according to word blocks (N = 1,010); Wilcoxon test for patients' and therapists' text (N = 1,010); M, mean; SD, standard deviation.

**Table 2** has the TCM patterns for the total text as well as for patients' and therapists' text. For patients' and therapists' text analyzed separately, *Relaxing* was the most prominent language pattern, followed by *Experiencing*. In the total text, *Relaxing* and *Connecting* were most frequent (for more detail, please view the **Supplementary Material**).

**Table 2:** TCM patterns in the total text and separately for patients and therapists.

Variables	Patients and Therapists	Patients	Therapists	Wilcoxon Patient vs. Therapists
	%	%	%	*p*
**Relaxing**	29.3	56.7	54.2	.490
**Reflecting**	22.9	11.1	14.2	.100
**Experiencing**	21.0	18.8	18.6	.733
**Connecting**	26.8	13.4	13.1	.900
**Part of TC**	41.9	14.6	18.0	.535

Relative frequencies of language patterns, and relative frequencies of therapeutic cycles (part of TC); segmentation at word block level, N = 1010; Wilcoxon tests for comparison of patients and therapists' text (N = 16).

### Therapeutic Process Measures

Well-being before therapy sessions was *M* = 5.57 (*SD* = 0.60, *N* = 16) and after therapy well-being averaged *M* = 6.21 (*SD* = 0.54, *N* = 16). A paired samples *t*-test revealed significantly higher well-being for patients after therapy (*t*(91) = -4.48, *p* < 0.001, *n* = 92). Ratings for well-being before and after therapy, and scores for therapy motivation are presented in the **Supplementary Material**.

Mean motivation to attend therapy was *M* = 6.17 (*SD* = 0.81). The therapists' ratings of the therapy process averaged *M* = 6.42 (*SD* = 0.73). Productiveness was rated *M* = 3.25 (*SD* = 0.77).

### Relationships Between Language Patterns and Session Ratings

To explore the relationships between TCM patterns (total text), and session ratings by patients, therapists, and observers, bivariate correlations were calculated (see **Table 3**). The relationship between patients' well-being before therapy and *Connecting* was negative (*r_s_* = −.56, *p <* 0.05). Another negative correlation was found for well-being before therapy, and the frequency of therapeutic cycles (*r_s_* = −.61, *p <* 0.05). The correlation between patients' well-being before the therapy sessions and positive emotional tone was positive (*r_s_* =.58, *p <* 0.05). There was a strong negative association of the frequency of therapeutic cycles and patients' well-being after session ratings (*r_s_* = −.79, *p <* 0.001). The therapists' session ratings were positively associated with positive emotional tone (*r_s_* =.70, *p <* 0.01). *Connecting* and the productiveness of the session as rated by the observer were not related (*r_s_* = 0.45, *p* = 0.08).

**Table 3:** Intercorrelations between session ratings and language variables and patterns in the total text.

Variables	1	2	3	4	5	6	7	8	9	10	11	12
Patient ratings												
1 Well-being before session												
2 Well-being after session	.63**											
3 Therapy motivation	.42	.53										
Therapist ratings												
4 Therapeutic process (per session)	.08	.17	.32									
Observer rating (KGPPS)												
5 Productiveness of the session	-.35	-.23	.23	.35								
Language variables and patterns												
6 Relaxing	.24	.25	.16	-.10	.19							
7 Reflecting	.16	.11	-.08	.25	-.41	-.67**						
8 Experiencing	.16	-.03	.06	-.31	-.17	-.52*	.23					
9 Connecting	-.56*	-.25	-.07	.23	.45+	.03	-.43	-.54*				
10 Positive emotional tone	.58*	.44	.43	.70**	.13	.07	.35	-.25	-.15			
11 Negative emotional tone	-.44	-.41	.00	.27	.13	-.54*	.37	-.03	.22	.01		
12 Therapeutic cycle	-.61*	-.79**	-.21	.02	.19	-.54	.14	.03	.40	-.25	.59*	

Language variables, language patterns, and session ratings of patients, therapists, and external raters; Spearman's rho rank correlations, N = 16 sessions. p < .05; **p < .01; + p < .10. N = 16.

## Discussion

The present study investigated micro-processes in a group psychotherapy based on a computerized analysis of transcripts of 16 consecutive therapy sessions with drug addicted forensic patients. We analyzed micro-processes in a group psychotherapy and described the frequency of different language patterns. Furthermore, the well-being of patients immediately prior and after therapy sessions was measured. Finally, the relations between language patterns and session ratings were analyzed.

Over 16 therapy sessions, therapists' and patients' proportions of text were similar. Other studies using the TCM reported higher proportions of text for patients (30, 34). The high proportion of therapists' text in the present study may be an indicator for the therapeutic style needed for the treatment of forensic patients (40). Emotional tone and abstraction was higher in the therapists' text, narrative style was higher in the patients' text. This is in line with the clinical finding that drug addicted patients often fail to verbally express or to regulate their emotions (41, 42). Patients “narrated” their own actions and those of other people rather than reflecting on them; in turn, the therapists actively asked for emotions that came along with the patients' narratives, or reflected on the meaning of what was said. When comparing the total text and patients' text, *Relaxing* was less frequent in the total text and *Connecting* more frequent than in patients' text. This may indicate that therapists completed the patients' narratives in order to create moments of insight in therapy. Forensic therapists should be able to identify and manage critical moments of therapy, helping their patients to assess critical personal experience, to shift from the negative into the positive, and to reflect about their own and others' negative and positive feelings. By doing this, a new therapeutic cycle may start (24).

Another aim of the present study was to analyze the relationship between the language patterns and session ratings. The focus was on the patients' well-being before and after the therapy session and therapy motivation. We found a negative relationship between the frequency of *Connecting* and patients' well-being before sessions, and a negative relationship between the frequency of therapeutic cycles and patients' well-being before and after sessions. Some level of discomfort or negative affect among the patients before group sessions started was related to the emergence of key moments in the sessions. Apparently, a low degree of (patients') well-being may be a precursor for therapeutic moments of insight to occur. Moreover, low levels of well-being after therapies were recorded in therapeutic cycles that followed a pattern of increasing emotional tone, followed by emotional insight and reflecting. Thus, well-being is not necessarily a consequence of key moments in therapy. On the contrary, in order for a therapeutic moment to qualify as a key moment that is likely to promote therapeutic change, it may be necessary to allow for feelings of malaise, discomfort, and trepidation. Psychotherapeutic progress requires work — hard work — on oneself, and the strength to tolerate frustration without giving up. Brenner (43) focused on investigating *Connecting* in forensic patients and neurotic patients. He stated that one quarter of the *Connecting* blocks in the forensic population were artifacts, i.e. emotionally toned words and abstract words were present in the very same scoring unit (word block), but semantically they were not connected to each other. By comparison, the neurotic population showed almost no artifacts. According to the Resonating Minds Theory and the principles of “deepen-and-provide” and “broaden-and-build”, the prototypical therapeutic cycle is characterized by a rise of positive and negative emotional tone in the *Connecting* phase in the middle of the cycle. In the subsequent *Reflecting* phase, a decline of negative emotional tone and a rise of positive emotional tone up to the end of the cycle are postulated. Taking these principles into account, the negative correlation between the frequency of therapeutic cycles and patients' well-being after the session is a rather unexpected result. It should be noted, however, that the operational definition of therapeutic cycles which was used in the present study relies on the sequence of the emotion-abstraction patterns and does not take into account the valence of emotions (shift from negative to positive emotional tone in the prototypical cycle). Thus, the negative correlation between well-being after the therapy session and therapeutic cycles may question whether the shift from “deepen-and-provide” to “broaden-and-build” (shift from negative to positive emotions) actually took place. Given the impaired emotional regulation often found in forensic patients with substance use disorders, it seems possible that the group participants were not able to shift from a negative to a positive emotional stance within a cycle, and/or they were unable to “hold” the positive emotional tone that was achieved within the therapeutic cycle till the end of the session. This interpretation would help to understand why an intensive therapeutic work, as denoted by a higher frequency of therapeutic cycles, was often followed by lower patients' well-being after the session.

Well-being before sessions was positively correlated with positive emotional tone. Thus, a patient's mood before a therapy session may be a predictor for positive emotional tone within the session. Besides, we found a strong positive relationship between positive emotional tone and the therapists' ratings of the therapeutic process after the session. Thus, the therapists considered therapeutic outcomes to be better when more positive emotional tone showed up in the session. This is in line with the findings of Chui and colleagues (10) who found that increase of positive affect was rated positively by therapists, for example in terms of higher engagement of the clients. A medium size correlation was found between *Connecting* and productiveness of the session. Further research is needed to corroborate this finding.

### Limitations

The group size was small, and a single-case study does not allow for generalization of the results. It was not possible to relate the micro-process data to broad outcome measures usually applied to forensic outcome studies (recidivism or reconviction rates, for example). Thus, we do not know the forensic outcomes of these patients. It is worthwhile to investigate other group therapies with forensic patients according to the TCM rationale, and to compare forthcoming results with the findings of the present study. Future studies may focus on the evolution of micro-processes over time. In the present study, single-item measures were used for well-being and therapy motivation. It is not wrong to use single items for measuring motivational and affective constructs (44–46), but the limitations associated with this approach are clear.

The method to identify language patterns focuses on the text level only. Therefore, nonverbal contents were not captured with the present analyses. Multiple correlations were not corrected for alpha inflation. Yet, the present findings can be considered to be a starting point for further research on therapeutic factors in forensic therapies.

### Conclusion

To conclude, the most insightful and productive moments in a therapy are not necessarily preceded or followed by immediate well-being of patients. Linguistic features of forensic group psychotherapies may be related to meaningful outcomes. In practical terms, it might be helpful to inform and support patients when insightful moments in therapy occur; patients should be made aware of the fact that insight may at times be painful if it is to promote cognitive and behavioral change. In critical moments, insight may come with impaired subjective well-being, and patients will need strong professional support to continue treatment.

## Data Availability Statement

The datasets generated for this study are available on request to the corresponding author.

## Ethics Statement

Ethical review and approval was not required for our study on human participants in accordance with the local legislation and institutional requirements. All the patients provided written consent before study entry.

## Author Contributions

TR and MF were responsible for the study protocol. MB and MF analyzed the data. MB, MF, TR, JB, and TK collaborated in writing the paper.

## Conflict of Interest

The authors declare that the research was conducted in the absence of any commercial or financial relationships that could be construed as a potential conflict of interest.

The reviewer EM declared a shared affiliation, with no collaboration, with one of the authors, JB, to the handling editor at the time of review.

## Footnotes

1 The present study draws on a previous paper by Bieg, Ross, Hoffmann, & Fontao (36). The analytic approach is different, with the present paper using the total text (instead of patients' text only) and focusing on patients' well-being before and after therapy. We consider the present paper complementary and equally valid, and adding substantially to the findings previously published.
