# Supplementary material for: Well-Being as a Precursor and Consequence of Micro-Processes in a Group Psychotherapy With Forensic Patients
Source: Front Psychiatry. 2020 Jun 3;11:409. doi: 10.3389/fpsyt.2020.00409 (PMC7283614; doi:10.3389/fpsyt.2020.00409)
Supplement: Supplementary file 1 [file DataSheet_1.docx]

Supplementary Material

# Supplementary Figures and Tables

*Supplementary Figure 1*. Patients’ ratings of well-being before and after therapy sessions, and therapy motivation.

*Supplementary Table 1*. Number of Therapeutic Cycles and the percentage of Therapeutic Cycles per session

|  | Number of  Therapeutic Cycles |  |  | Percentage of Therapeutic Cycles per session |
| --- | --- | --- | --- | --- |
| Therapy session |  |  |  |  |
| 1 | 4 |  |  | 35.0 |
| 2 | 3 |  |  | 33.3 |
| 3 | 5 |  |  | 47.5 |
| 4 | 5 |  |  | 49.2 |
| 5 | 5 |  |  | 34.4 |
| 6 | 4 |  |  | 37.1 |
| 7 | 5 |  |  | 36.4 |
| 8 | 2 |  |  | 41.8 |
| 9 | 7 |  |  | 48.6 |
| 10 | 4 |  |  | 24.5 |
| 11 | 4 |  |  | 54.9 |
| 12 | 6 |  |  | 40.9 |
| 13 | 7 |  |  | 63.6 |
| 14 | 4 |  |  | 54.9 |
| 15 | 4 |  |  | 29.8 |
| 16 | 5 |  |  | 37.7 |

*Note.* Segmentation according to word blocks (*N* = 1010). Percentage of Therapeutic Cycles denotes the proportion of text segments identified as parts of Therapeutic Cycles relative to the total number of text segments per session. An example: If a session consists of 64 word blocks, 16 of which are part of a Therapeutic Cycle, the percentage of Therapeutic Cycles in that session is 16/64 = 25%.

*Supplementary Figure 2.* Graphic illustration of the text analysis according to the Therapeutic Cycles Model (TCM)


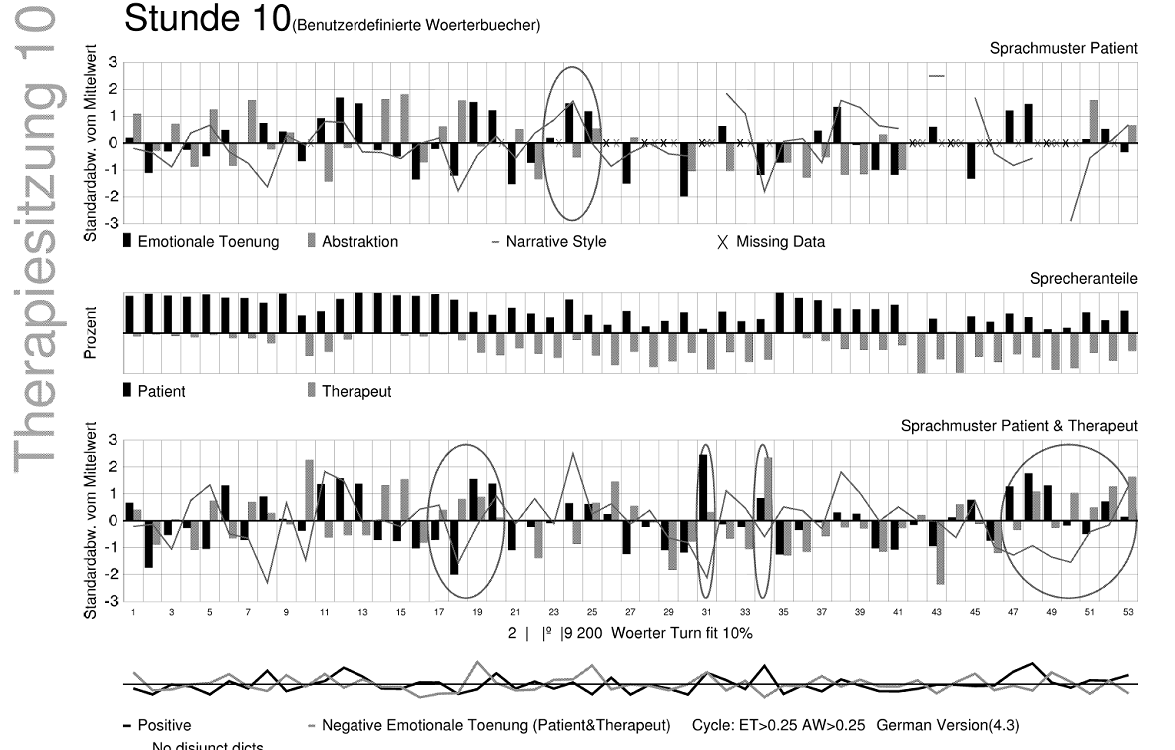


**Ex**

**Rx**

**Rf**

**Co**

**Co**

**Co**

# Samples from transcript of therapy session 10

Session 10, word block 2: Relaxing (Rx)

*<P1> (P15:) dass ich dann hiermit auch so ein bisschen mache, können wir nachher noch mal drüber reden. ansonsten geht's gut (E:WB)*

*<P1> (P05:) ja ich fühle mich so körperlich schon ziemlich gut, zur Zeit ist halt mein Kopf irgendwie Totalschaden so*

*<P2> (T1:) wie kommen Sie jetzt darauf - -*

*<P1> (P05:) na wie soll ich das sagen, ich krieg irgendwie schon vieles gut hin und so aber ich denk dann halt immer dass ich mach dann halt von mir selber aus voll die Hektik und so ich denk ich schaffe das nicht und so und genauso gestern mit dem A. wie wir beim Sport waren da wollte unbedingt irgendwas machen so mit so Seilen und so was eigentlich das kann ich gar nicht machen und ich denk dann ha ja zieh das durch und so und das musst du hinkriegen keine Ahnung das sage ich selber zu mir so die ganze Zeit und na ja dann habe ich mir vorgenommen irgendwie*

*noch meinem Bruder einen Brief zu schreiben. ich weiß nicht ich wollte da auch noch mit Ihnen reden wie und was ich*

*genau da schreibe*

*<P2> (T1:) mmh*

*<P1> (P05:) und lass mir auch Zeit damit, aber ich möchte das unbedingt machen und so, wenigstens sagen was ich irgendwie will und damit (E:WB)(2)(S:WB) alles und er dann vielleicht normal wird vielleicht oder auch nicht, kommt*

*drauf an wie der das will und ha ja muss ich mir halt noch einfallen lassen wie ich das formuliere oder keine Ahnung.*

*dann gucke ich halt noch was beim Besuch am Wochenende rauskommt so mit meinen Eltern noch mal versuchen auch zu reden, denen halt zu sagen wie es mir halt zur Zeit geht und so, mal gucken wie die das so aufnehmen und so*

English translation

<P1> (P15 :) that I can do a bit with this, we can talk about it again later. otherwise it's fine (E: WB)

<P1> (P05 :) yes I feel pretty good physically, at the moment my head is kind of total damage

<P2> (T1 :) how do you get this now - -

<P1> (P05 :) well how can I say that, I somehow manage a lot well, but then I always think that I get hectic and I think I can't do it. Just yesterday with A as we were exercising he wanted to do something with ropes and I really can't do it and then I think, ha, I´ve got to finish this and I´ve got to get this done, I have no idea I say that to myself all the time and well then I somehow made up my mind to write a letter to my brother. I don't know I wanted to talk to you about how and what I write to him.

<P2> (T1 :) mmh

<P1> (P05 :) and take my time with it, but I really want to do it, at least say what I want (E: WB) (2) (S: WB) and then he may or may not be normal, it depends on what he wants and ha yes I have yet to come up with how I put it, I have no idea.

Then I'll just watch what happens when I visit on the weekend so try to talk to my parents again, just tell them how I'm doing at the moment, let's see how they take it.

Session 10, word block 10: Reflecting (Rf)

*<P1> (P09:) [… ]aber das wird die Zeit einfach bringen wenn es ich kann mich ja nur beweisen in dem Sinne jetzt ja dass sie sieht dass das nicht so aufgesetzt war oder so jetzt kann es wirklich mal erprobt werden in den vierzehn Tage, fünfzehn Tage wo ich daheim bin (E:WB)*

*<P2> (T2:) ja wobei ich finde schon dass es ist ja am Montag habe ich noch mal hingeguckt in Ihren Bericht und ich frage mich da schon also was könnten Sie denn eigentlich tun was für Ihre Freundin oder Partnerin als Beweis der also anerkannt werden würde also was müssten Sie tun damit Ihre Freundin merkt dass Sie tatsächlich anders sind? und da habe ich schon große Zweifel ob Sie das überhaupt leisten können. was wollen Sie denn tun? dass Ihre Freundin einen anderen Eindruck bekommt als das was sie aktuell meint. wissen Sie was ich meine?*

*<P1> (P09:) mmh*

*<P2> (T2:) Sie können eigentlich gar nichts tun weil das geht ja das ist ja allein ihr Ding im Moment*

*<P1> (P09:) am Wochenende habe ich zu ihr gesagt das ist ihr Kopfgeficke muss ich sagen so das ist nicht meins, von meiner Seite her habe ich alles getan ich werde nach wie vor für sie da sein (E:WB)*

English translation

<P1> (P09 :) […] but that will come with time I can only prove myself in the sense that now yes that she sees that it was not set up or so now it can really be tried out in the fourteen, fifteen days when I am at home (E: WB)

<P2> (T2 :) yes, although I think yes, on Monday I looked again at your report and I am already wondering what you could do for your girlfriend or partner as evidence that could be accepted so what would you have to do to make your girlfriend realize that you are actually different? and I have big doubts if you can do that at all. What do you want to do that your girlfriend gets a different impression than what she currently thinks. Do you know what I mean?

<P1> (P09 :) mmh

<P2> (T2 :) There is really nothing you can do because that’s all up to you at the moment

<P1> (P09 :) at the weekend I said to her that this is her headfucking, I have to say this is not my cup to tea, from my side I`ve done everything I will still be there for her (E: WB)

Session 10, word block 39: Experiencing (Ex)

*<P2> (T2:) […] für den Bruder, für sich, für andere Personen. was versprechen Sie sich davon also Sie für sich wissen das ja eigentlich. oder der Beweis dafür dass Sie jetzt der Coolere sind von Ihnen beiden also wenn das Ihre Überzeugung ohnehin ist dann müssten Sie das ja jetzt nicht*

*<P1> (P05:) nein ich das hat für mich eigentlich jetzt gar nicht so groß damit zu tun wer jetzt der Coolere von uns beiden ist aber ich hab den ja wie ich hab halt mit dem versucht normal zu reden und so weiter und auch aggressiv und alles Mögliche und wenn ich zu dem sage ich rufe dich an und ich rufe ein paar Sekunden später an und der hebt nicht ab dann*

*<P2> (T1:) dann sind Sie stinkig - Wut*

*<P1> (P05:) ja und ich weiß nicht und dann macht er es halt einfach so locker und jetzt will ich dem halt einfach die*

*paar Sätze hinschreiben und den irgendwie dann wo der auch überhaupt nicht erwartet, wo der gar nicht dass so was von*

*mir kommt und so*

*<P2> (T1:) ihn mal brieflich abwatschen*

*<P1> (P05:) ja*

*<P2> (T2:) und das kennt er doch eigentlich von Ihnen oder? dass er abgewatscht wird, dass ein Brief kommt oder / ist das die neue Variante aber (E:WB)(39)(S:WB)*

English translation

<P2> (T2 :) […] for the brother, for yourself, for other people. What do you expect from it…so you actually know that yourself. The proof that you are now the cooler of the two of you, so if that is your conviction anyway, then you wouldn’t have to do that now

<P1> (P05 :) no I actually don't have that much to do with who is the cooler of us now but I tried to talk to him like that and so on and sometimes aggressively and all sorts of things and when I say that I call you and I call a few seconds later and he doesn't answer

<P2> (T1 :) then you´re pissed off - anger

<P1> (P05 :) yes and I don't know and then he just plays me so easily and now I just want that write down a few sentences and something he doesn't expect at all, something he doesn't expect that to come from me

<P2> (T1 :) lambast him by letter

<P1> (P05 :) yes

<P2> (T2 :) and he actually knows that from you, right? that you lambast him, that a letter comes or / is this the new variant (E: WB) (39) (S: WB)

Session 10, word block 20: Connecting (Co)

*<P2> (T2:) ist der denn neu der Traum? oder ist das ein alter. ist es eine alte Gestalt oder ist es Ihnen erstmals so sichtbar*

*<P1> (P05:) einmal jetzt erste*

*<P2> (T2:) erste mal*

*<P1> (P05:) mal kenne ich nicht*

*<P2> (T1:) so wie Kopf war er so leuchtend man sah kein Gesicht na?*

*<P1> (P05:) ja*

*<P2> (T1:) und spricht flüsternd*

*<P1> (P05:) und ich hab auch nicht verstanden was der sagt gar nichts*

*<P2> (T1:) also murmelt so*

*<P1> (P05:) ja und dann wollte ich ja weggehen die ganze Zeit so damit es mich in Ruhe lässt und aber durch das Flüstern und so musste ich wieder zurücklaufen, ich konnte*

*<P2> (T1:) / was*

*<P1> (P05:) ja. es hat mich halt zurückgezogen so - -*

*<P2> (T2:) ja was ist das für eine Gestalt, der lockt, der verführt? was macht er denn mit Ihnen? ärgert er Sie? ist es einfach nur reizvoll? - die Gestalt ist größer als Sie oder?*

*<P1> (P05:) schon auf jeden Fall und ist voll ruhig geblieben, stand nur auf einem Platz und hat die ganze Zeit geflüstert - -*

*<P2> (T2:) so ein erleuchtetes Gesicht gehabt - -*

*<P1> (P05:) es wollte auch auf jeden Fall nicht erkannt werden oder so*

*<P2> (T2:) muss man sich das wie so eine Mönchskapuze oder so vorstellen, die er dann über dem Kopf hat und wo das*

*Gesicht dann leuchtete oder?*

*<P1> (P05:) ha ja*

*<P2> (T2:) langer Mantel (E:WB)(20)*

English translation

<P2> (T2 :) is the dream new? or is that an old one? is it an old shape or is it visible to you for the first time

<P1> (P05 :) now first

<P2> (T2 :) first time

<P1> (P05 :) I don't know times

<P2> (T1 :) the head it was so bright you couldn't see a face, hm?

<P1> (P05 :) yes

<P2> (T1 :) whispers

<P1> (P05 :) and I didn't understand what he says, nothing at all

<P2> (T1 :) mumbles

<P1> (P05 :) yes and then I wanted to go away all the time so that it leaves me alone and but the whisper made me run back again, I could

<P2> (T1 :) / what

<P1> (P05 :) yes. it just pulled me back like this - -

<P2> (T2 :) yes, what kind of shape is it that attracts, seduces? what is he doing to you? does he annoy you? is it just appealing? - the shape is bigger than you, isn´t it?

<P1> (P05 :) definitely and it has remained completely calm, stood only in one place and whispered all the time - -

<P2> (T2 :) had such an enlightened face - -

<P1> (P05 :) it definitely didn't want to be recognized

<P2> (T2 :) you have to think of it as a monk's hood or something, which he then pulls over his head and then the face lit up, right?

<P1> (P05 :) ha yes

<P2> (T2 :) long coat (E: WB) (20)

Session 10, word block 26: Connecting (Co)

*<P2> (T2:) […] mehreren, das ist nicht nur eine sondern sind häufig auch wirklich in einer*

*Person versammelt mehrere drinnen wo Sie sind ist völlig klar, was Sie von der Person wollen eigentlich auch. was Sie*

*sich von der Person nehmen ist ja auch bezeichnend dann, dass Sie da einverleiben das hat ja irgendwie auch schon*

*eine richtige Kraft finde ich, also Ihre eigene Ihre Motivation die wird ja auch deutlich. Sie wollen an das Herz*

*von der Person würde ich jetzt mal von außen sehen, auch ziemlich wertfrei*

*<P1> (P05:) ja wozu, um die kaputt zu machen oder was*

*<P1> (P15:) / / / (Pause 00:00:12)*

*<P2> (T2:) vielleicht war das das Wesentliche von der Person, das Gesicht hat sie nicht, man kann sie nicht verstehen, sonst ist sie nicht erkennbar - - Sie sagen die Person ist Ihnen wichtig gleichzeitig wissen Sie auch eine andere Person die Ihnen wichtig wäre wäre würde es nie zum Problem werden zum Thema werden. es betrifft dann schon denke ich wichtige Person*

*<P1> (P05:) ja ich hab halt schon oft dran gedacht dass es mein Bruder sein könnte aber ich will das eigentlich gar nicht dass er das war (E:WB)(26)(S:WB)*

English translation

<P2> (T2 :) […] several, that is not just one but often really many in one

person and where they are is entirely clear, what they want from a person is clear, too. What they take away from the person is also significant and what they incorporate from that person is powerful so their motivation is clear. They want straight to the heart of the person, I would say, no judgment.

<P1> (P05 :) yes, what for, to break them or what

<P1> (P15 :) / / / (pause 00:00:12)

<P2> (T2 :) maybe that was the essence of the person, she has no face, you cannot understand her, you cannot identify her - - you say the person is important, at the same time you know another person important to you would never allow this to become a problem. It already affects that important person

<P1> (P05 :) yes I've often thought that it could be my brother but I don't really want him to be that person (E: WB) (26) (S: WB)

Session 10, word block 31: Connecting (Co)

*<P1> (P05:) ungefähr halt*

*<P2> (T1:) mmh?*

*<P1> (P05:) ungefähr halt oder*

*<P2> (T1:) mmh oder wo man merkt an*

*irgendwas das ist doch was Ähnliches*

*<P2> (T2:) ich glaube das Problem für Sie vielleicht ist einfach ein bisschen an der Stelle dass Sie sich möglicherweise einfach auch ein bisschen schlechtes Gewissen machen, ich weiß nicht mal warum also Sie dürfen denke ich ganz entspannt drauf gucken was da passiert ist also da ist ja nichts konkretes passiert. es hat Ihnen vielleicht Angst gemacht irgendwie, kann schon sein aber die Frage ist wenn Sie da ein bisschen entspannter drauf gucken, was ist denn da Schlimmes da gewesen also dieser*

*<P2> (T1:) ja im Traum ist ja alles erlaubt*

*<P2> (T2:) dessen Gesicht Sie eigentlich gerne gesehen hätten und der einfach weitermurmelt, einfach nur erleuchtet sein möchte, das reicht ihm einfach dass er bereit ist das Herz sich rausreißen zu lassen, ist immer noch erleuchtet und Sie*

*wollen ja nicht nur das Herz also ich denke da wird schon so ein Thema deutlich*

*<P1> (P05:) mmh. - - na ja dann können es ja nur meine Eltern sein oder (E:WB) (31)*

English translation

<P1> (P05 :) just about

<P2> (T1 :) mmh?

<P1> (P05 :) just about, isn´t it

<P2> (T1 :) mmh or how do you notice that

something is similar to something else

<P2> (T2 :) I think the problem for you perhaps may be that you just feel a little guilty, I don't even know why, so I think you can relax and watch what happens there, well nothing happened in particular. It may have scared you somehow, that may well be, but the question is, if you look at it a little more relaxed, what was the terrible thing there, so this..

<P2> (T1 :) yes everything is allowed in a dream

<P2> (T2 :) whose face you would actually like to see and who just keeps on mumbling, just wants to be enlightened, he´s fine with having his heart torn out, is still enlightened and they not only want the heart so I think there´s a pattern visible

<P1> (P05 :) mmh. - - well then it can only be my parents (E: WB) (31)
